# Effectiveness of combinations of active compression-decompression cardiopulmonary resuscitation, impedance threshold devices and head-up cardiopulmonary resuscitation in adult out-of-hospital cardiac arrest: A systematic review

**DOI:** 10.1016/j.resplu.2024.100760

**Published:** 2024-09-09

**Authors:** Shona E. Main, David B. Sidebottom, Charles D. Deakin, James Raitt, Helen Pocock, Julian Hannah, James O.M. Plumb

**Affiliations:** a Thames Valley Air Ambulance, United Kingdom; b Hampshire and Isle of Wight Air Ambulance, United Kingdom; c University Hospital Southampton NHS Foundation Trust, United Kingdom; d South Central Ambulance Service NHS Foundation Trust, United Kingdom; e Integrative Physiology and Critical Illness Group, Clinical and Experimental Sciences, Faculty of Medicine, University of Southampton, United Kingdom; f Perioperative and Critical Care Theme, NIHR Southampton Biomedical Research Centre, Southampton NHS Foundation Trust, United Kingdom; g University Hospitals Dorset, United Kingdom; h Frimley Park Hospital, United Kingdom; i Södertälje Sjukhus, Stockholm, Sweden

**Keywords:** Out of hospital cardiac arrest, Cardiopulmonary resuscitation, Impedance threshold device, Active compression-decompression, Head-up CPR (HUP-CPR), Advanced life support

## Abstract

**Objective:**

This review summarises the current evidence base for combinations of neuroprotective CPR adjuncts (active compression-decompression chest compressions, impedance threshold devices, and head-up positioning) during out-of-hospital cardiac arrest.

**Methods:**

A systematic search (PROSPERO registration CRD42023432302) was performed in English on MEDLINE, EMBASE, and the Cochrane Library in August 2023, and repeated in February 2024. All randomised and observational studies (not abstracts) reporting on any combination of the aforementioned CPR adjuncts were included. Papers were screened independently by two researchers, with a third reviewer acting as tiebreaker. Out-of-hospital, non-traumatic, cardiac arrests in patients >18 years were eligible for inclusion. Risk of bias was assessed using the Risk of Bias 2 tool and the Newcastle-Ottawa scale.

**Results:**

Eight of 1172 unique articles identified in the initial searches were included, with five randomised controlled trials and three observational studies. No randomised trial investigated a bundle of all three interventions. All randomised controlled trials were at intermediate or high risk of bias. Neurologically favourable survival was greater in patients treated with an impedance threshold device and active compression-decompression CPR when compared to standard CPR (8.9% vs 5.8%, *p* = 0.019) in the largest existing randomised trial. Conflicting results were found in observational studies comparing the complete neuroprotective bundle to standard CPR.

**Conclusions:**

This review was limited by small study numbers and overlapping samples, which precluded a meta-analysis. Limited data suggests that combinations of adjuncts to improve cerebral perfusion during CPR may improve survival with favourable neurological outcome. A randomised controlled trial is required to establish whether combining all three together results in improved outcomes.

## Introduction

Maintaining cerebral perfusion during cardiopulmonary resuscitation (CPR) is vital to achieve good neurological outcome following out-of-hospital cardiac arrest (OHCA).^1^ Maximising cerebral arterial pressure and minimising central venous and intrathoracic pressure optimises cerebral perfusion pressure (CePP) and therefore cerebral oxygen delivery during CPR.^2^, ^3^ Through these mechanisms, a number of CPR adjuncts including active compression-decompression (ACD) CPR, impedance threshold devices (ITDs), and head-up positioning (HUP) may improve CePP and have the potential to improve survival and neurological outcome following cardiac arrest.^2^, ^3^, ^4^

ACD-CPR generates a negative intrathoracic pressure during the decompression phase of chest compressions via a suction cup applied to the anterior chest wall. ITDs augment this effect by restricting the influx of air into the lungs that would otherwise occur when a negative intrathoracic pressure is generated, thereby enhancing and prolonging negative intrathoracic pressure.^5^ In combination, these adjuncts optimise venous return and ventricular filling during the diastolic phase of CPR, thereby improving mean arterial pressure and CePP.^3^, ^4^, ^5^ HUP, which can be delivered via an automated self-elevating backboard (ACE-CPR), may additionally optimise cerebral blood flow during CPR by increasing venous drainage from the head and neck and decreasing intracranial pressure.^6^, ^7^ The synergistic effects of these three combined interventions, henceforth referred to as the neuroprotective CPR bundle, further optimises CePP during CPR in animal and human cadaver models.^8^

Despite promising preclinical data and observational research spanning over 20 years,^5^, ^9^, ^10^ these devices, when used in isolation, have generally failed to show an improvement in survival and in particular, neurologically intact survival in clinical trials.^11^ More recently however, findings from a number of cohort studies suggest that using combinations of these devices may improve outcome from OHCA.^12^, ^13^

The urgent need to improve the evidence base for neuroprotective CPR has recently been highlighted in *Resuscitation*,^14^ while optimisation of CPR is an International Liaison Committee on Resuscitation (ILCOR) research priority.^15^ A recent UK pre-hospital modified Delphi study ranked interventions beyond current ALS guidelines as one of the top three focuses for research over the next five years.^16^ This systematic review is, to the authors’ knowledge, the first to summarise the current evidence base for combinations of neuroprotective CPR adjuncts during OHCA. It is additionally aligned with priority setting work with patients and family members,^17^ where the urgent need to identify on-scene interventions to improve outcomes from OHCA has been identified.^16^

## Methods

This is a systematic review of strategies to optimise CPR during OHCA, involving ACD-CPR, ITDs, and HUP/ACE-CPR. The protocol was pre-registered on the PROSPERO database (CRD42023432302).^18^ The review is reported in accordance with the PRISMA statement (2020) and associated PRISMA-S extension for literature searches.^19^, ^20^

### Searches

The peer-reviewed search strategy was reviewed by an information specialist and targeted any combination of keywords and synonyms relating to the cardiopulmonary resuscitation adjuncts specified in the research question. Searches were executed by the information specialist in English on MEDLINE, EMBASE, and The Cochrane Library (Cochrane Database of Systematic Reviews, Cochrane Central Register of Controlled Trials (CENTRAL), Cochrane Methodology Register) on August 10, 2023. Databases were searched from January 1, 1990. Additionally, the WHO international clinical trials registry platform was searched for planned or ongoing clinical trials. The search strategies are available in full online.^18^ The search was repeated on February 15, 2024 to identify any additional eligible articles published after the date of the original search.

### Types of participants

Patients with OHCA were eligible for inclusion. Exclusion criteria were paediatric patients (<18 years), patients where resuscitation was not attempted, and traumatic cardiac arrest. Animal and human cadaveric studies were also excluded.

### Types of study

All RCTs and observational studies were eligible for inclusion (including cohort, case–control, case series, and individual case reports). Abstracts alone were not included.

### Types of interventions

Eligible studies included any combination (2 or more) of ACD-CPR, an ITD, and HUP/ACE-CPR.

ACD-CPR was defined as an intervention where a device is attached by negative pressure to the sternum, allowing active physical compression and decompression of the chest. An ITD was defined as equipment which restricts airflow into the lungs during the recoil or decompression phase of cardiopulmonary resuscitation, thereby lowering intrathoracic pressure. HUP-CPR was defined as any intervention where cardiopulmonary resuscitation is performed with the head and thorax actively elevated from a resting position.

### Outcomes

The pre-defined co-primary outcomes were good neurological outcome by any measure and survival to hospital discharge. Secondary outcomes included return of spontaneous circulation (ROSC) and survival to hospital admission.

### Selection, screening and data extraction

The output of all searches was imported into Rayyan review management software.^21^ Automated duplicate screening was performed; all identified duplicates were manually verified by a reviewer. Two reviewers, blinded to each other’s decisions, independently screened remaining studies against the pre-defined eligibility criteria, first by title and abstract and subsequently by full text review. After unblinding, disagreements were resolved by a third reviewer where consensus could not be achieved.

Two reviewers extracted data into a standardised data-collection form. Extracted data was cross-checked by the other reviewer and disagreements were resolved by consensus. Extracted data included study design, study methodology, patient demographics and baseline data (age, gender, rhythm, witnessed arrest, bystander interventions, duration prior to emergency services arrival, duration of cardiac arrest), interventions and control conditions, study outcomes, and results. Data is available from the authors on request.

### Data synthesis and risk of bias assessment

Data was summarised and explored in tabular form. Numbers of participants and events, odds ratios, and *p* values were extracted for all outcomes where reported. While a meta-analysis was planned, this was abandoned due to limited, overlapping, and heterogeneous data.

The risk of bias in included studies was assessed by consensus between two reviewers (SEM, DBS). Randomised controlled trials were assessed using the Risk of Bias 2 (RoB2) tool while observational trials were assessed using the Newcastle-Ottawa Scale as laid out in the Cochrane handbook.^22^

## Results

**Fig. 1:**
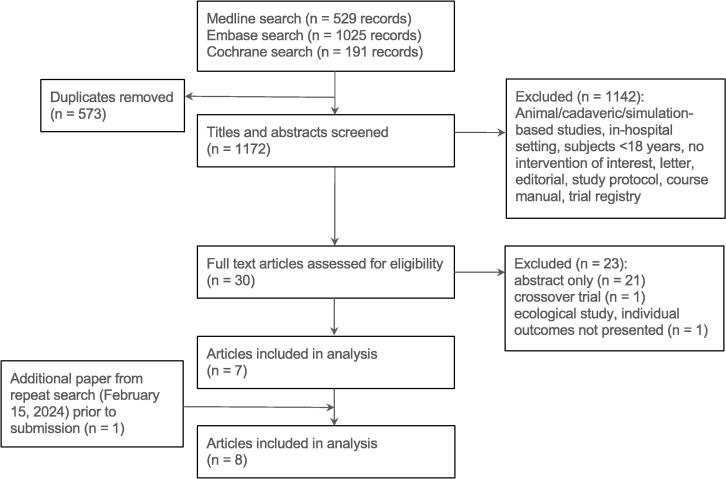
Summary of the results of the search strategy.

Overall, 1172 unique articles were identified, of which eight were included in the final analysis (Fig. 1),^12^, ^13^, ^4^, ^23^, ^24^, ^25^, ^26^, ^27^ Some participants in five of the papers selected for inclusion overlapped with at least one of the other papers. These papers were included due to the limited amount of literature identified and are clearly marked in all tables. One additional paper meeting inclusion/exclusion criteria was identified on the repeat search (February 15, 2024) and was subsequently included in the analysis.

Characteristics of included studies are summarised according to population, intervention, comparison, and outcomes in Table 1. Included studies collected data spanning from 1997 to 2020 across France, Germany, and the USA. Three RCTs (*n* = 2944) compared standard CPR (S-CPR) with ACD in combination with an ITD,^23^, ^25^, ^26^ two RCTs (*n* = 421) compared ACD-only CPR to ACD in combination with an ITD,^4^, ^24^ two observational studies (with overlapping participant groups) compared S-CPR with the neuroprotective CPR bundle using propensity matching,^12^, ^13^ and one before/after observational study (*n* = 2162) compared ACD in combination with an ITD with the neuroprotective bundle.^27^

**Table 1:** Study characteristics.

Study	Data source	Country	Data collection	Study Design	Blinding	Main inclusion/exclusion criteria.	Standard/control management.	Intervention(s)	Primary Outcome
ACD vs. ACD+ITD									
Plaisance, 2000^4^	−	France	1997	Randomised controlled trial	Clinician & assessor blinded to ITD (sham or functional)	OHCA in patients ≥18 years old with non-traumatic aetiology. Patients with hypothermia, terminal illness, or BLS duration >30 mins were excluded.	ERC and AHA guidelines.	(1) ITD or (2) ACD plus ITD. In addition, endotracheal intubation, femoral arterial line, femoral central venous catheter for monitoring.	ETC02, diastolic blood pressure, coronary perfusion pressure and time to ROSC
Plaisance, 2004^24^	−	France	1999–2000	Randomised controlled trial	Clinician blinded to ITD (sham or functional)	OHCA in patients ≥18 years old with non-traumatic aetiology. Patients with hypothermia, an obvious non-survivable injury, terminal illness, or BLS duration >30 mins were excluded.	ERC 2000 guidelines. All patients included were intubated and ventilated with a portable pressure-cycle ventilator.	(1) ACD plus sham ITD or (2) ACD plus active ITD.	24 h survival
S-CPR vs ACD+ITD									
Wolcke, 2003^23^	−	Germany	1999–2002	Randomised controlled trial	Neither clinician nor assessor blinded	OHCA with presumed cardiac aetiology. Patients with hypothermia, a DNR order, terminal illness, or downtime >15 mins prior to initiation of CPR were excluded.	ERC 1998 guidelines and AHA 2000 guidelines. All patients were intubated prior to randomisation.	ACD (CardioPump device) plus ITD (ResQValve).	1 h survival post witnessed arrest
Aufderheide, 2011^25^	Resuscitation Outcomes Consortium Prehospital Resuscitation Impedance Valve and Early Versus Delayed Analysis	USA	2005–2010	Randomised controlled trial	Assessor blinded	OHCA in patients ≥18 years old with presumed cardiac aetiology. Patients with a DNR order, recent sternotomy, or obvious signs of death were excluded.	AHA 2005 guidelines.	ACD (CardioPump device) plus ITD (ResQValve). The ITD could be used in combination with a facemask or advanced airway.	Modified Rankin Scale ≤3 at hospital discharge
Frascone, 2013^26^	Resuscitation Outcomes Consortium Prehospital Resuscitation Impedance Valve and Early Versus Delayed Analysis	USA	2005–2010	Secondary analysis of RCT (Aufderheide 2011)	Assessor blinded	OHCA in patients ≥18 years old of any non-traumatic aetiology. Patients with a DNR order, recent sternotomy, or obvious signs of death were excluded.	AHA 2005 guidelines.	ACD (CardioPump device) plus ITD (ResQValve). The ITD could be used in combination with a facemask or advanced airway.	Modified Rankin Scale ≤3 at hospital discharge
ACD+ITD vs. neuroprotective CPR bundle (ACD, ITD, and ACE-CPR)									
Pepe, 2019^27^	International Device Assisted Controlled Sequential Elevation CPR Registry	USA	2014–2017	Before/after study (prospective)	N/A	All consecutive OHCA	AHA guidance as of 2015 with addition of LUCAS device and an ITD	Standard management plus 20 degrees head elevation (reverse Trendelenberg) in addition to a package of CRM training.	Clinical safety and feasibility of bundle
Moore, 2022^12^	International Device Assisted Controlled Sequential Elevation CPR Registry	USA	2019–2020^a^	Registry study (prospective)	N/A	OHCA in patients ≥18 years old. Patients currently in prison were excluded.	Control (S-CPR) patients were extracted from three randomised controlled trials including the ROC trial above (Aufderheide 2011). AHA 2005 guidelines onwards.	ACD (CardioPump device) or LUCAS 2.0/3.0, ITD (ResQValve), and EleGARD patient positioning system (stepwise elevation of the head and thorax to 22 cm and 9 cm respectively).	Survival to hospital discharge
Bachista, 2004^13^	International Device Assisted Controlled Sequential Elevation CPR Registry	USA	2019–2021	Registry study (prospective)	N/A	OHCA with non-shockable rhythms in patients ≥18 years old of non-traumatic origin	Control (S-CPR) patients were extracted from two randomised controlled trials including the ROC trial above (Aufderheide 2011).	ACD (ResQPUMP or LUCAS), ITD (ResQPOD), and EleGARD patient positioning system (stepwise elevation of the head and thorax from 12/8cm, respectively to 24/12 cm, respectively over 2 min)	Survival to hospital discharge

All patients were blinded to treatment allocation by nature of cardiac arrest.

OHCA=Out of hospital cardiac arrest; ACD=Active compression-decompression; ICD Impedence threshold device; ERC=European Resuscitation Council; AHA=American Heart Association; S-CPR=Standard CPR; ACE-CPR=Automated controlled-elevation CPR; LUCAS=Lund University Cardiopulmonary Assist System; CRM=Crew resource management.

a ACE-CPR cases collected between these dates, control cases collected from previous randomised controlled trials.

### Risk of bias

An intermediate risk of bias was identified in two of the five RCTs included, while a high risk of bias was identified in the remaining three (Table 2). The most common reasons for higher risk of bias were issues with the randomisation process and risk of selective reporting of outcomes. The observational studies were rated as low risk of bias (Table 3).

**Table 2:** Risk of bias assessments for included randomised controlled trials (*n* = 5).

Study	Experimental	Comparator	Outcome	Weight	Domain 1: Randomisation process	Domain 2: Deviations from the intended interventions	Domain 3: Missing outcome data	Domain 4: Measurement of the outcome	Domain 5: Selection of the reported result	Overall risk of bias
Plaisance, 2000	ACD+ITD CPR	ACD CPR	Number discharged from hospital	1						
Wolcke, 2003	ACD+ITD CPR	S-CPR	CPC and OPC at hospital discharge	1						
Plaisance, 2004	ACD+ITD CPR	ACD CPR	CPC at hospital discharge	1						
Aufderheide, 2011	ACD+ITD CPR	S-CPR	MRS at hospital discharge	1						
Frascone, 2013	ACD+ITD CPR	S-CPR	CPC, OPC and MRS at 1 year	1						

ACD=Active compression-decompression; ITD=Impedence threshold devce; CPR=Cardiopulmonary resuscitation; S-CPR=Standard CPR; CPC=Cerebral Performance Category; OPC=Overall Performance Category; MRS=Modified Rankin Score.

**Table 3:** Risk of bias for the included observational trials (*n* = 3) using the Newcastle Ottawa Scale.

Study ID	Experimental	Comparator	Outcome	Selection	Comparability	Outcome	Overall	Risk of Bias
Pepe, 2019	ACD+ITD CPR	Neuroprotective CPR bundle (ACD, ITD, and ACE-CPR)	Clinical safety and feasibility of bundle	****	*	***	8/9	Low
Moore, 2022	S-CPR	Neuroprotective CPR bundle (ACD, ITD, and ACE-CPR)	Survival to hospital discharge	***	**	***	8/9	Low
Bachista, 2004	S-CPR	Neuroprotective CPR bundle (ACD, ITD, and ACE-CPR)	Survival to hospital discharge	***	**	***	8/9	Low

ACD=Active compression-decompression; ITD=Impedence threshold devce; CPR=Cardiopulmonary resuscitation; ACE-CPR=Automated controlled elevation CPR; S-CPR=Standard CPR.

### Summary measures

Measures of effect are presented in Table 4. A neurologically favourable outcome varied from 1.1% to 10.3% in the S-CPR group and between 5.0% and 13.6% when ACD was combined with an ITD.^12^, ^13^, ^23^, ^25^, ^26^ A neurologically favourable outcome was higher with ACD+ITD compared with S-CPR in one RCT (OR 1.42 [95% CI 1.04–3.27]),^26^ and not significantly different in another (13.6% with ACD+ITD vs. 10.3% with S-CPR, *p* = 1.0).^23^ A neurologically favourable outcome was more likely with the neuroprotective bundle when compared with S-CPR in one observational study (OR 3.87 [95% CI 1.27–11.78]),^13^ and was not significantly different in another.^12^

**Table 4:** Study outcomes.

Study	Neurologically favourable outcome					Survival to discharge					Survival to hospital/ICU admission					ROSC				
	S-CPR (%)	ACD (%)	ACD+ITD (%)	ACE-CPR (%)	Significance test	S-CPR (%)	ACD (%)	ACD+ITD (%)	ACE-CPR (%)	Significance test	S-CPR (%)	ACD (%)	ACD+ITD (%)	ACE-CPR (%)	Significance test	S-CPR (%)	ACD (%)	ACD+ITD (%)	ACE-CPR (%)	Significance test
ACD vs. ACD+ITD																				
Plaisance, 2000^4^	−	Not reported	Not reported	−	Not reported	−	1/10 (10.0)	1/11 (9.1)	−	*p* = 0.9	−	Not reported	Not reported	−	Not reported	−	2/10 (20)	4/11 (36)	−	*p* = 0.04
Plaisance, 2004^24^	−	8/200 (4.0)^a^	10/200 (5.0)^a^	−	OR 1.26 (95% CI 0.49–3.27); *p* = 0.63	−	8/200 (4.0)	10/200 (5.0)	−	OR 1.26 (95% CI 0.49–3.27); *p* = 0.63	−	57/200 (28.5)	79/200 (39.5)	−	OR 1.64 (95% CI 1.08–2.49); *p* = 0.02	−	77/200 (38.5)	96/200 (48)	−	OR 1.48 (95% CI 0.99–2.19); *p* = 0.056
S-CPR vs ACD+ITD																				
Wolcke, 2003^23^	11/107 (10.3)^b^	−	14/103 (13.6)^b^	−	OR 0.8 (95% CI 0.6–3.0); *p* = 1.0	14/107 (13.1)	−	19/103 (18.4)	−	OR 1.3 (95% CI 0.6–3.0); *p* = 0.41	34/107 (32)^c^	−	53/103 (51)^c^	−	OR 2.1 (95% CI 1.2–3.9); *p* = 0.006	40/103 (37)	−	57/103 (55)	−	OR 2.0 (95% CI 1.1–3.5); *p* = 0.016
Aufderheide, 2011^25^	47/813 (5.8)^b^	−	75/840 (8.9)^b^	−	0.019	80/813 (9.8)	−	104/840 (12.8)	−	*p* = 0.12	216/813 (26.6)	−	237/840 (28.2)	−	Not significant^d^	324/813 (39.9)	−	343/840 (40.8)	−	Not significant^d^
Frascone, 2013^26^	75/1335 (5.6)^b^	−	110/1403 (7.8)^b^	−	OR 1.42 (95% CI 1.04–1.95), *p* = 0.027	134/1335 (10.0)	−	165/1403 (11.8)	−	*p* = 0.16	376/1335 (28.2)	−	431/1403 (30.7)	−	Not significant^d^	537/1335 (40.2)	−	591/1403 (40.1)	−	Not significant^d^
ACD+ITD vs. neuroprotective CPR bundle (ACD, ITD, and ACE-CPR)																				
Pepe, 2019^27^	−	−	Not reported	Not reported	Not reported	−	−	Not reported	Not reported	Not reported	−	−	Not reported	Not reported	Not reported	−	−	144/806 (17.9)	464/1356 (34.2)	*p* < 0.001
Moore, 2022^e^^12^	35/860 (4.1)^a^^/^b	−	−	13/222 (5.9)^a^^/^b	OR 1.47 (95% CI 0.76–2.82)	58/860 (6.7)	−	−	21/222 (9.5)	OR 1.44 (95% CI 0.86–2.44)	Not reported	−	−	Not reported	Not reported	282/860 (32.8)	−	−	74/222 (33.3)	OR 1.02 (95% CI 0.75–1.49
Bachista, 2024^e^^13^	4/353 (1.1)^a^^/^b	−	−	15/353 (4.2)^a^^/^b	OR 3.87 (95% CI 1.27–11.78)	10/353 (2.8)	−	−	27/353 (7.6)	OR 2.84 (95% CI, 1.35–5.96)	Not reported	−	−	Not reported	Not reported	Not reported	−	−	Not reported	Not reported

ROSC=Return of Spontaneous Circulation; CPR=Cardiopulmonary resuscitation; S-CPR=Standard CPR; ACD=Active compression-decompression; ITD=Impedence threshold devce; ACE-CPR=Automated controlled elevation CPR.

a Modified Rankin Score ≤3.

b Cerebral Perfusion Category 1 or 2.

c One hour after ICU admission.

d Value not reported by paper.

e Results after propensity score matching.

Survival to discharge ranged between 2.8% and 13.1% in the S-CPR groups,^13^, ^23^ between 5.0% and 18.4% where ACD was combined with an ITD,^23^, ^24^ and was 7.6% and 9.5% in the two observational studies reporting this measure for the neuroprotective bundle.^12^, ^13^ Only one, observational study reported a higher survival to discharge with the neuroprotective bundle when compared to S-CPR (2.8% vs 7.6%, OR 2.84 [95% CI 1.35–5.96]),^13^ while all other studies reporting this measure did not find a significant difference between the control and intervention arms.

Four articles reported survival to hospital/ICU admission, of which one demonstrated a significant difference between groups.^23^ A significant difference in ROSC between S-CPR and ACD combined with an ITD in one RCT (37% versus 55%, *p* = 0.016).^23^ A subsequent large RCT did not observe a difference in ROSC between S-CPR and ACD combined with an ITD (39.9% versus 40.8%, non-significant).^25^ No significant difference in ROSC was observed in the single study comparing S-CPR with the neuroprotective bundle where this was reported.^12^

## Discussion

This systematic review provides a comprehensive overview of the evidence for combinations of neuroprotective CPR adjuncts during OHCA. A limited number of studies were identified, mostly at high risk of bias. The largest RCT was terminated early due to funding constraints and did not meet the target sample size deemed necessary according to the interim analysis.^25^ No published RCT was identified comparing any control group to the complete neuroprotective bundle (ACD, ITD and HUP-CPR).

Aufderheide and colleagues performed a prospective, randomised, assessor blinded, multicentre trial comparing ACD+ITD CPR with S-CPR.^25^ They found survival to hospital discharge with a good neurological outcome was 5.8% (47/813) in the control group versus 8.9% (75/840) in the intervention group (*p* = 0.019, OR 1.58 [95% CI 1.07–2.36]). Importantly, in a secondary analysis this finding was robust to relaxation of the inclusion criteria from only patients with a presumed cardiac aetiology to all non-traumatic OHCAs (*p* = 0.027, OR 1.42 [95% CI 1.04–1.95]),^26^ increasing the robustness of these findings. Neither analysis observed a difference in ROSC. While a previous, small RCT comparing S-CPR to ACD+ITD CPR was not sufficiently powered to assess a difference in neurologically favourable outcome, it did identify a higher rate of ROSC, survival to 1 h after ICU admission (*p* = 0.006, OR 2.1 [95% CI 1.2–3.9]), and survival at 24 h within the intervention group.^23^ It is therefore notable that all RCTs comparing S-CPR to ACD+ITD CPR found significant differences in the primary outcome for which they were statistically powered. However, the relevance of these studies to present day is limited by technical and non-technical advances in cardiac arrest management, as well as demographic changes, that have taken place in the two decades since the start of participant recruitment.

Bachista and colleagues recently performed a propensity matched observational study using data from a U.S. national registry of patients with non-shockable rhythms who received the neuroprotective bundle (ResQPUMP; ZOLL Medical, Chelmsford, MA or LUCAS; Stryker Medical, Kalamazoo, MI, ResQPOD; ZOLL Medical, and EleGARD Patient Positioning System; AdvancedCPR Solutions, Edina, MN),^13^ compared to control data from patients managed with S-CPR in two historical large RCTs.^11^, ^25^ In unadjusted analyses, the likelihood of survival and survival with good neurological function was significantly higher in the group treated with the neuroprotective bundle (OR 3.09 [95% CI 1.64–5.81]). After propensity score and time interval matched analyses, the neuroprotective bundle was still associated with higher odds of survival with good neurological function (OR 3.87 [95% CI 1.27–11.78]). There were no statistically significant differences the rate of ROSC between the two groups.

This large and recent study provides a strong signal to the potential benefits of neuroprotective CPR, while noting that the benefits were particularly pronounced when the intervention was initiated within 15 min of the emergency call and that it was a non-randomised study. This is supported by earlier work suggesting a dose response relationship between time to initiation of the bundle and survival with favourable neurological outcome,^12^ and has physiological validity in that optimisation of CePP is likely to have the greatest effect when implemented prior to catastrophic irreversible anoxic cerebral injury. The control groups were however recruited from different geographical sites and were not contemporaneous and the authors of this study acknowledge this as a meaningful limitation.^12^, ^13^, ^28^

### Strengths and limitations

This systematic review included a robust methodology with involvement of an information specialist in the design and execution of the comprehensive searches, as well as independent article screening and dual-assessor data extraction. The primary outcome was patient-centred.

The primary limitation of this review is the scarcity of evidence. One large RCT dominates the randomised studies, while heterogeneity of the control and intervention groups makes comparisons difficult. Data on the complete neuroprotective bundle originated entirely from observational studies, which while of generally good quality, are inherently at high risk of selection bias. A number of articles included overlapping samples in either the intervention or comparison groups, as highlighted in the results section. A further limitation was the timespan of data collection; included data spanned 1997 to 2020 and therefore multiple iterations of AHA/ERC guidelines in addition to demographic changes that may affect the prognostic features of patients presenting with OHCA.

## Conclusions

This systematic review demonstrates that clinical evidence for the benefits of the neuroprotective CPR bundle is scarce, but that combinations of CPR devices that act to enhance cerebral blood flow during CPR may improve outcome following OHCA. It remains unclear which specific endpoints (e.g. ROSC, survival, survival with good neurological outcome), if any, may be improved by a neuroprotective CPR bundle during OHCA. Equipoise exists to support an appropriately powered randomised controlled trial with a patient-centred outcome to further investigate the efficacy of the neuroprotective CPR bundle during OHCA.

## Ethics approval and consent to participate

Not applicable as a systematic review.

## Availability of data and materials

Links to the study protocol and search string can be found in the text and on the PROSPERO registry. The search string will become visible in PROSPERO on publication. All data generated during this study are included in this published article.

## Funding sources/sponsors

The authors provided their time in kind for this work.

## CRediT authorship contribution statement

**Shona E. Main:** Writing – review & editing, Writing – original draft, Methodology, Investigation, Formal analysis, Data curation. **David B. Sidebottom:** Writing – review & editing, Writing – original draft, Methodology, Investigation, Formal analysis, Data curation. **Charles D. Deakin:** Writing – review & editing, Supervision, Methodology, Conceptualization. **James Raitt:** Writing – review & editing, Supervision, Methodology, Conceptualization. **Helen Pocock:** Writing – review & editing, Conceptualization. **Julian Hannah:** Writing – review & editing, Conceptualization. **James O.M. Plumb:** Writing – review & editing, Writing – original draft, Validation, Supervision, Formal analysis, Conceptualization.

## Declaration of competing interest

The authors declare that they have no known competing financial interests or personal relationships that could have appeared to influence the work reported in this paper.
